# Temporal Repression of Core Circadian Genes Is Mediated through EARLY FLOWERING 3 in *Arabidopsis*

**DOI:** 10.1016/j.cub.2010.12.013

**Published:** 2011-01-25

**Authors:** Laura E. Dixon, Kirsten Knox, Laszlo Kozma-Bognar, Megan M. Southern, Alexandra Pokhilko, Andrew J. Millar

**Affiliations:** 1 Centre of Systems Biology at Edinburgh, University of Edinburgh, Edinburgh, Midlothian EH9 3JD, UK; 2 Institute of Molecular Plant Sciences, University of Edinburgh, Edinburgh, Midlothian EH9 3JH, UK; 3 Institute of Plant Biology, Biological Research Center, H-6726 Szeged, Hungary; 4 Department of Biology, University of Warwick, Coventry CV4 7AL, UK

## Abstract

The circadian clock provides robust, ∼24 hr biological rhythms throughout the eukaryotes. The clock gene circuit in plants comprises interlocking transcriptional feedback loops, reviewed in [1], whereby the morning-expressed transcription factors CIRCADIAN CLOCK-ASSOCIATED 1 (CCA1) and LATE ELONGATED HYPOCOTYL (LHY) repress the expression of evening genes, notably *TIMING OF CAB EXPRESSION 1* (*TOC1*). *EARLY FLOWERING 3* (*ELF3*) has been implicated as a repressor of light signaling to the clock [2, 3] and, paradoxically, as an activator of the light-induced genes *CCA1* and *LHY* [4, 5]. We use *cca1-11 lhy-21 elf3-4* plants to separate the repressive function of ELF3 from its downstream targets *CCA1* and *LHY*. We further demonstrate that ELF3 associates physically with the promoter of *PSEUDO-RESPONSE REGULATOR 9* (*PRR9*), a repressor of *CCA1* and *LHY* expression, in a time-dependent fashion. The repressive function of ELF3 is thus consistent with indirect activation of *LHY* and *CCA1*, in a double-negative connection via a direct ELF3 target, *PRR9*. This mechanism reconciles the functions of ELF3 in the clock network during the night and points to further effects of ELF3 during the day.

## Highlights

► ELF3 is a regulator of *TOC1*, *PRR9*, *GI*, and *PRR7* gene expression ► Repression by ELF3 is genetically separable from repression by LHY and CCA1 ► ELF3 physically associates with the promoter of *PRR9* in a time-dependent manner

## Results

### Mutual Regulation of *ELF3* and *CCA1/LHY* Expression

**Figure 1:**
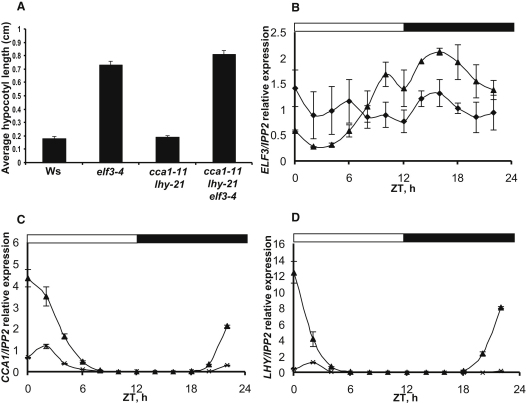
ELF3 Affects Clock Outputs and Clock Genes Hypocotyl measurements of 7-day-old seedlings are shown as an average hypocotyl length (Wassilewskija [Ws] n = 12, *elf3-4* n = 18, *cca1-11 lhy-21* n = 19, and *cca1-11 lhy-21 elf3-4* n = 23), with the error being represented as a standard error of the mean (SEM) (A). Data are representative of two biologically independent experiments. qPCR measurements are shown of RNA levels for *ELF3* in Ws wild-type plants (filled triangles) and *cca1-11 lhy-21* double mutants (filled diamonds) (B), *CCA1* (C) and *LHY* (D) in Ws (filled triangles), and *elf3-4* mutants (crosses). Data are all normalized against *IPP2* expression [25]. Graphs are an average of two to three biologically independent experiments, with normalized data being used to generate SEM error bars. Seedlings were grown in 12:12 white light:dark cycles and sampled every 2 hr from Zeitgeber time (ZT) = 0. ZT = 0 is defined as the time of lights-on. See also Figures S1 and S2.

Hypocotyl growth is a circadian output and can be used as an indicator of clock function [6]. *elf3-4* seedlings show abnormally elongated hypocotyls as the clock-controlled repression of hypocotyl growth is lost in these plants [7, 8]. To study the interaction between *ELF3* and *CCA1/LHY*, we examined hypocotyl length in loss-of-function mutant backgrounds. Seedlings were grown under short day conditions (6:18 hr light:dark cycles) for 6 days, and hypocotyl length was assessed on day 7. The wild-type ecotype, Wassilewskija (Ws), and *cca1-11 lhy-21* seedlings showed hypocotyls of similar length, whereas *cca1-11 lhy-21 elf3-4* showed a phenotype very similar to *elf3-4* seedlings (Figure 1A). This suggests that the *elf3-4* mutant effect on aberrant growth of hypocotyls does not require the LHY and CCA1 transcription factors. Imaging of rhythms in delayed chlorophyll fluorescence (see Figure S1 available online) showed that, like *elf3-4* mutants, *cca1-11 lhy-21 elf3-4* plants were arrhythmic for this physiological marker in constant light.

*CCA1* and *LHY* RNA expression levels were shown to be very low in *elf3* mutant seedlings, suggesting a mechanism for their arrhythmia [5]. We confirmed this through quantitative PCR (qPCR) analysis on 7-day-old seedlings under 12:12 white light:dark (LD) cycles (Figure 1) or transferred from 12:12 red LD to constant light (LL; Figure S2). The high amplitude of *CCA1* and *LHY* expression rhythms in wild-type (100-fold to 1000-fold in LD, 10-fold in LL) collapsed in the *elf3-4* plants, which became arrhythmic in LL. Transcript analysis under LD was more informative. The low-amplitude rhythm in both *CCA1* and *LHY* transcripts (reaching at most 15% of wild-type peak level, Figures 1C and 1D; 40% of wild-type peak, Figures S2A and S2C) showed that the clock's morning functions were severely impaired in the *elf3-4* mutant, though a rhythm could still be driven by the LD cycle. *ELF3* RNA levels had a lower-amplitude rhythm in the wild-type (at most 10-fold in LD), whereas in *cca1-11 lhy-21* mutants, *ELF3* RNA showed little rhythmicity under LD and arrhythmia under LL (Figure 1B and Figure S2F). Circadian control of *ELF3* expression [5] requires the morning loop components CCA1 and LHY. ELF3, in turn, regulates these clock genes and gates entrainment signals [2].

### ELF3 Is a Key Repressor of Core Circadian Genes

**Figure 2:**
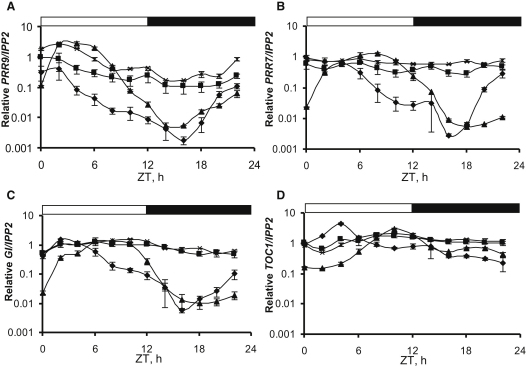
ELF3 Regulates the Expression of Core Circadian Genes qPCR measurements of RNA levels for *PRR9* (A), *PRR7* (B), *GI* (C), and *TOC1* (D) normalized against *IPP2* and between replicates in Ws (filled triangles), *elf3-4* (crosses), *cca1-11 lhy-21* (filled diamonds), and *cca1-11 lhy-21 elf3-4* (filled squares). Graphs are an average of three biologically independent experiments, each containing triplicate samples. Normalized data were used to generate SEM error bars. Seedlings were grown and sampled as in Figure 1. See also Figure S3.

An evening loop, involving at least *TOC1* and *GIGANTEA* (*GI*), is proposed to generate the short-period rhythms observed in *lhy cca1* double mutants [9]. Through the comparison of clock gene expression in Ws, *elf3-4*, *cca1-11 lhy-21*, and *cca1-11 lhy-21 elf3-4* plants, we aimed to test the role of *ELF3* in the proposed evening loop. Plants were grown under 12:12 LD cycles for 6 days, sampled on day 7, and tested for expression of *PRR9*, *PRR7*, *GI*, and *TOC1* (Figure 2; Figure S3). In *cca1-11 lhy-21* plants, the evening genes (*TOC1* and *GI*) showed an early-morning peak of high amplitude (Figures 2C and 2D). This is in agreement with previously published data [9–11] and also supports the hypothesis that CCA1 and LHY act to repress evening gene expression in the early morning. In the double mutant, *PRR9* showed a lower amplitude rhythm, probably because of the loss of activation of expression by CCA1 and LHY (Figure 2A). The *elf3-4* mutant showed a lower amplitude rhythm in gene expression for all measured genes, with notably higher levels (over 10-fold increase compared to wild-type) of *PRR9*, *PRR7*, and *GI* expression in the night, as reported in [12] for *GI*, as well as slightly higher nighttime expression of *TOC1*. The aberrant gene expression continued into the early morning, when CCA1 and LHY should be active in the wild-type (Figures 1C and 1D). Such results are consistent with a combination of indirect and direct mechanisms, whereby CCA1 and LHY repress evening gene expression in the morning (Zeitgeber time [ZT] 0–4, where ZT = 0 is defined as the time of lights-on) and ELF3 represses many genes at night (ZT 12–20), before CCA1 and LHY are expressed. From this it could be expected that the *cca1-11 lhy-21 elf3-4* triple mutant would show high expression of certain clock genes throughout the LD cycle. This was not observed. Instead, in the triple mutant, all genes were expressed at intermediate levels, without strong responses to the ongoing LD. *PRR9* and *PRR7* expressions were higher than in *cca1-11 lhy-21* but lower than in *elf3-4*. Evening genes (*TOC1* and *GI*) lost the early peak observed in *cca1-11 lhy-21*, but then had the higher nighttime expression characteristic of *elf3-4*. This suggests that ELF3 influences the circadian network at more than one point and thus affects both morning and evening loops.

### ELF3 Binds In Vivo to the Promoter of *PRR9* in the Early Night

**Figure 3:**
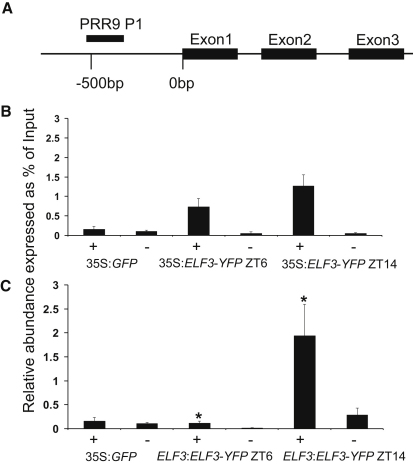
ELF3 Binds In Vivo to the Promoter of *PRR9* in the Early Night, but Not during the Day (A) Schematic of the *PRR9* genomic region tested. The black bar indicates the specific region amplified from ChIP DNA by primer set P1. (B and C) Chromatin of 3-week-old plants was immunoprecipitated using either no antibody (−) or anti-GFP antibody (+). Resultant DNA extracted from 35S::*GFP* (B and C), 35S::*ELF3::YFP* (B), and *ELF3::ELF3::YFP* (C) plants was analyzed by qPCR. Each signal is expressed as a percentage of the signal in nonimmunoprecipitated DNA (input) extracted from the same tissue sample. Data represent the mean of at least six samples taken from three independent ChIP experiments. Error bars represent the SEM. Student's t test showed that only *ELF3::ELF3::YFP* had significantly different chromatin association between ZT = 6 and ZT = 14, marked with ^∗^p < 0.05. See also Figure S4.

Because *ELF3* shows some sequence homology with transcription factors [13], we investigated whether ELF3 physically associates with circadian-controlled promoters. Chromatin immunoprecipitation (ChIP) experiments were conducted using transgenic plants that expressed an *ELF3::YFP* fusion protein from either the native *ELF3* promoter or the 35S*CaMV* promoter. We also used 35S::*ELF4::YFP* to investigate whether ELF3 and ELF4 act on the same promoters. *EARLY FLOWERING 4* (*ELF4*) is a circadian-controlled gene that shows similar gene expression patterns and clock phenotypes to *ELF3* [14]. ELF3 and ELF4 were both able to associate with the *PRR9* promoter (Figures 3B and 3C; Figure S4). However, when ELF3 was expressed from its native promoter, it showed time-dependent affinity for the *PRR9* promoter, being bound at ZT = 14 but not significantly (by Students t test) at ZT = 6 (Figure 3C). ELF3's apparently rhythmic association with the *PRR9* promoter and the increased *PRR9* expression observed in the *elf3-4* mutant suggest that ELF3 acts as one of the repressors of *PRR9* gene expression. Association of ELF3 with the *PRR7* promoter was weak, because it was detected only in the 35S::*ELF3::YFP* plants (Figure S4). Association of ELF4 with *PRR7* was comparable to results for *PRR9* (Figure S4). Testing 1.3 kbp of sequences upstream of the ATG codon of *CCA1* did not reveal any ELF3 or ELF4 association (data not shown), although this promoter fragment is sufficient for rhythmic transcription [15]. However, derepression of the *PRR9* promoter is sufficient to explain low levels of *CCA1* and *LHY* expression in the *elf3-4* mutant (Figures 1C and 1D), because PRR9 is a known repressor of *CCA1* and *LHY* [16]. The promoter regions required for rhythmic expression of *PRR5*, *TOC1*, and *GI* were also tested, and ELF3 and ELF4 were not found to associate with these (data not shown), suggesting that ELF3 is involved in the regulation of their expression indirectly.

### A Combination of Repressors Is Required for the Control of Circadian-Regulated Light Responses

**Figure 4:**
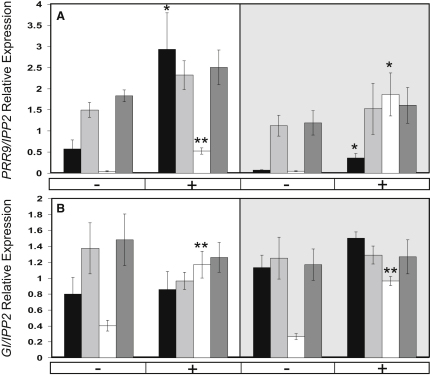
ELF3 Is Required for the Control of Circadian-Regulated Light Responses in *GI* and *PRR9* Acute light induction of *PRR9* (A) and *GI* (B) gene expression was measured by qPCR in Ws (black bars), *elf3-4* (light gray bars), *cca1-11 lhy-21* (white bars), and *cca1-11 lhy-21 elf3-4* (dark gray bars). Seedlings were grown for 5 days under white-light 12:12 LD cycles and released into continuous dark from ZT = 12 on day 5. On day 6, samples were either treated with (+) or without (−) a white-light pulse (20 min, 80 μmol m^−2^ s^−1^) 1 hr before sampling on the predicted day at ZT = 30 (white background) and on the predicted night at ZT = 38 (gray background). Error bars indicate the SEM from 4–6 samples. Student's t test was used to compare treated and untreated samples within a time point and genotype. For clarity, only treated samples that differ significantly from their control are marked with ^∗^p < 0.05 or ^∗∗^p < 0.005.

In order to investigate the regulation of light signaling via ELF3, a 20 min white-light pulse was applied to seedlings entrained in 12:12 white LD cycles and released into darkness. *PRR9* and *GI* were specifically investigated because they have both been implicated in light signaling to the clock [16, 17], showed misregulation of gene expression in the *elf3-4*, *cca1-11 lhy-21*, and *cca1-11 lhy-21 elf3-4* mutants, and represented the morning and evening loops of the circadian network. Wild-type plants showed strong light induction of *PRR9* (Figure 4A). In *cca1-11 lhy-21* double mutants, the expression levels of *PRR9* were very low, and a clear acute response to light was observed, which was as large or larger than that in Ws during the predicted night, ZT = 38 (Figure 4A). In *elf3-4* and *cca1-11 lhy-21 elf3-4* seedlings, *PRR9* had a higher level of basal expression in the night, consistent with Figure 2A and with ELF3's function as a repressor of gene expression in the dark. Little change in expression was observed following a light pulse at either predicted day ZT = 30 or night ZT = 38 (Figure 4A). Notably, the *PRR9* expression level was not maximal compared to peak levels (Figure 2A), suggesting that another factor is involved in the gating of light responses in the dark. *GI* expression was not light responsive at these times in Ws and showed light induction in *cca1-11 lhy-21*, but not in *elf3-4* or the triple mutant (Figure 4B). This again indicates that ELF3 affects clock gene expression in darkness, that ELF3 still controls clock genes in *cca1-11 lhy-21* seedlings, and that some repressive functions remain in the triple mutant.

## Discussion

This work tests the possibility that *ELF3* acts as an activator of *CCA1* through both the investigation of the transcriptional loops with which *ELF3* is involved and the determination of whether ELF3 protein can associate with DNA. We show that *ELF3* has repressive effects on several clock genes. The observed activation of *CCA1* in *elf3-4* mutants can be explained consistently with ELF3's repressive function by a double-negative effect via *PRR9*, the repressor of *CCA1* and *LHY* [18]. ELF3 protein associates with the *PRR9* promoter (Figure 3). In *elf3-4*, the levels of *PRR9* are high, so the repression of *CCA1* and *LHY* is greater. However, the high expression of evening genes *GI* and *TOC1* in *elf3-4* mutants cannot simply be explained by low levels of CCA1 and LHY, because this high baseline was not observed in *cca1-11 lhy-21* mutants.

To investigate the role of *ELF3* independently of the influence of *CCA1* and *LHY*, we generated *cca1-11 lhy-21 elf3-4* plants. These plants have a growth phenotype similar to the *elf3-4* plants (Figure 1A). *cca1-11 lhy-21 elf3-4* mutants show high basal levels of clock gene expression in the dark period of 12:12 LD cycles, as in *elf3-4*, but do not show the characteristic early peaks of *PRR7*, *GI*, and *TOC1* expression observed in *cca1-11 lhy-21* (Figure 2). This high level of gene expression in the dark is also observed in the acute light pulse response data set (Figure 4). Thus, through comparison of the *cca1-11 lhy-21* and *cca1-11 lhy-21 elf3-4* data, it seems that *ELF3* allows rhythmicity in the *cca1-11 lhy-21* double mutant. It also suggests that there may be another, normally redundant, factor, which is able to take the role of *CCA1/LHY* in the early morning (ZT 0–4) and repress the expression of circadian genes. This function is not observed in the *cca1-11 lhy-21* double mutant because the component is still being repressed by ELF3.

Association with the *PRR9* promoter provides a mechanism for ELF3's direct (*PRR9*) and indirect (*CCA1/LHY*) effects on the clock network. The fact that *ELF3* affects the clock network beyond the times when ELF3 is detected at the *PRR9* promoter is consistent with the known complexity of the clock circuit (Figure 2; Figure 3). Our current mathematical model of the *Arabidopsis* clock includes repression of *PRR9* by an evening gene and assigns this role to *TOC1* based on the known repression of *PRR9* expression in *TOC1*-overexpressing plants [19]. It will now be important to understand the interaction of TOC1 and ELF3.

ELF3 is known to have a number of binding partners, including the red-light photoreceptor PHYB, the ubiquitin E3-ligase COP1, and clock-related proteins GI, SVP, and CCA1, suggesting that ELF3 may function in large signaling complexes. In this setting, ELF3 could participate in protein degradation [20] or transcriptional control through transcriptional complexes or histone and/or other chromatin modifications. Such an interpretation is supported by the mild phenotypic effect of the ELF3 overexpressor on the clock network [3] compared to the severe effect of the mutant; the ELF3 protein is required for correct clock function, but its level might not be so important.

This work identifies ELF3 as repressing gene expression of clock components, resulting in widespread effects on the clock gene network. Thus, ELF3 is essential for the normal operation of the circadian transcriptional feedback loops in light-grown plants, as reported in dark-grown seedlings [21]. The mechanism of ELF3 action presented here links *ELF3* directly to the circadian network.

## Experimental Procedures

### Construction of Multiple Mutant Lines and Transgenic Plants

To create the *cca1-11 lhy-21 elf3-4* triple mutant, we crossed the *cca1-11 lhy-21* [22] double mutant to *elf3-4* [6]. In the F2 progeny, individuals with long hypocotyls were selected and verified as homozygous *elf3-4* mutants. These plants were then screened for *cca1-11* and *lhy-21* mutations. For details on the molecular markers used for genotyping, see Table S1.

The *ELF3* promoter and the *ELF3* and *ELF4* coding sequences (CDS) were amplified by PCR from wild-type Ws genomic DNA by PCR primers with added restriction sites to facilitate cloning. The sequence of primers and the corresponding restriction sites are provided in Table S2. The amplified fragments were cloned in pBlueScript SK plasmids and verified by sequencing. The *ELF3* promoter fragment contained 2695 nucleotides upstream of the start codon of the *ELF3* gene and included the full 5′ untranslated region. The *ELF3* and *ELF4* CDS fragments included the full coding sequence but not the translational termination codons. The *35S:PHYA-YFP* pPCVB812 binary vector has been described [23]. The *PHYA* cDNA in *35S:PHYA-YFP* pPCVB812 was replaced with the *ELF3* or *ELF4* CDS fragments, resulting in *35S:ELF3-YFP* pPCVB812 and *35S:ELF4-YFP* pPCVB812. Then the *35S* promoter in *35S:ELF3-YFP* was replaced by the *ELF3* promoter fragment, which yielded *ELF3:ELF3-YFP* pPCVB812. The binary vectors containing the gene constructs described above were transferred to *Agrobacterium tumefaciens* GV3101 cells. The constructs were transformed into wild-type Ws (*ELF4* construct) and *elf3-4* mutant plants (*ELF3* constructs) by the floral-dip method [24]. Primary transformant plants were isolated based on resistance to Basta herbicide. Ten to 15 independent transgenic lines were produced for each combination of construct and host plant. Lines carrying a single copy of the transgene were selected based on the segregation of Basta resistance and were used for experiments.

### Plant Materials and Growth Conditions

All plant lines are in the Ws ecotype. Surface sterilized seeds were stratified for 4 days in the dark at 4°C before being grown under cool-white fluorescent tubes (70–100 μmol m^−2^ s^−1^) in LD cycles at constant 22°C. All plants were grown on 1% agar Murashige-Skoog (MS) plates. Photoperiod light conditions were either short day (SD) 6:18 or standard 12:12, as shown in figures. ZT = 0 is defined as the last dark:light transition before measurements start.

### Analysis of Gene Expression

For LD time courses, approximately 70 seedlings per sample were harvested for each genotype into 1 ml of RNAlater solution (Ambion). Samples were taken at 2 hr intervals starting at ZT = 0. Total RNA was extracted (QIAGEN RNeasy kit, 74106) according to manufacturer's instructions. cDNA was synthesized from 1 μg of total RNA, and random hexamer primers were supplied with the Fermentas cDNA synthesis kit. cDNA was diluted 1:5 in RNase-free dH_2_O, and qPCR plates (LightCycler 480 multiwell plate 384, Roche) were set up using a Tecan Freedom EVO robot controlled by EVOware standard software with Master Mix containing SYBR Green (Roche), gene-specific primers at 3 μM, and RNase-free dH_2_O. The qPCR was conducted in triplicate in a Roche LightCycler 480 controlled by LightCycler 480 SW1.5 software. Transcript levels were normalized to the control transcript *IPP2* [25] and were normalized between replicates.

All presented measurements are an average of three independent experiments. Gene-specific primer pairs are listed in Table S2.

### Measurement of Hypocotyl Length

Plants were grown under SD (6:18 LD) white-light (70–100 μmol m^−2^ s^−1^) photoperiod conditions on MS and 1% agar plates for 6 days, and hypocotyls with centimeter ruler were imaged using a digital camera. Measurement of hypocotyl length was performed by ImageJ (http://rsb.info.nih.gov/ij/), with hypocotyl length being defined as from V in hypocotyls-cotyledon formation to hypocotyls-root junction.

### Chromatin Immunoprecipitation

ChIP was carried out as previously described [26], with the following modifications: seedlings were grown for 3 weeks in 12:12 LD cycles and harvested at either ZT = 6 or ZT = 14; crosslinking with 1% formaldehyde was carried out under a vacuum for a total of 30 min; and samples were resuspended in 4 ml of ChIP dilution buffer and split into four samples. Chromatin was immunoprecipitated using anti-GFP (Clontech). ChIP DNA was analyzed by qPCR on an LC480 (Roche) using SYBR Green Master Mix (Roche). Relative quantities were calculated as a percentage of the input DNA for each sample. Primer pairs for each region tested are listed in Table S2 and were designed to cover the promoter regions previously shown to be sufficient for normal expression in promoter:LUC reporters [14, 15].

## Supplemental Information

Document S1. Four Figures and Two Tables
