# Supplementary material for: Temporal Repression of Core Circadian Genes Is Mediated through EARLY FLOWERING 3 in Arabidopsis
Source: Curr Biol. 2011 Jan 25;21(2):120–5. doi: 10.1016/j.cub.2010.12.013 (PMC3028277; doi:10.1016/j.cub.2010.12.013)
Supplement: Document S1. Four Figures and Two Tables [file mmc1.pdf]

Current Biology, Volume *21*

Supplemental Information

Temporal Repression of Core Circadian Genes Is Mediated through EARLY FLOWERING 3 in *Arabidopsis*

Laura E. Dixon, Kirsten Knox, Laszlo Kozma-Bognar, Megan M. Southern, Alexandra Pokhilko, and Andrew J. Millar

A

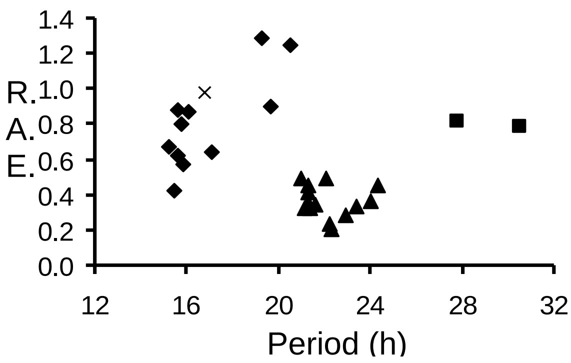

B

Genotype	n	rhythmic	% rhythmic
Ws wild type	16	14	88
cca1-11 lhy-21	16	11	69
elf3-4	16	1	6
cca1-11 lhy-21 elf3-4	16	2	13

Figure S1 (Related to Figure 1). Seedlings Carrying the *elf3-4* Mutation Are Arrhythmic in Constant Light
Delayed chlorophyll [S1] fluorescence from 7-day old seedlings grown in 96-well multiwell plates was measured on an automated TopCount scintillation counter over 4 days in constant light. Fluorescence collected for 2 seconds per well with no count delay, from n=16 wells, each well containing 3-4 plants, combining two biologically independent experiments. Ws (filled triangles), *elf3-4* (cross), *cca1-11 lhy-21* (filled diamonds) and *cca1-11 lhy-21 elf3-3* (filled squares). Rhythmicity was assessed using FFT-NLLS analysis [S2] in the BRASS v3 software package [S3]. Rhythm robustness is represented as A) estimated period (hours) versus the relative amplitude error (RAE, where low RAE values represent robust rhythmicity and RAE=1 represents arrhythmia) and B) the number and percentage of rhythmic samples from both experiments.

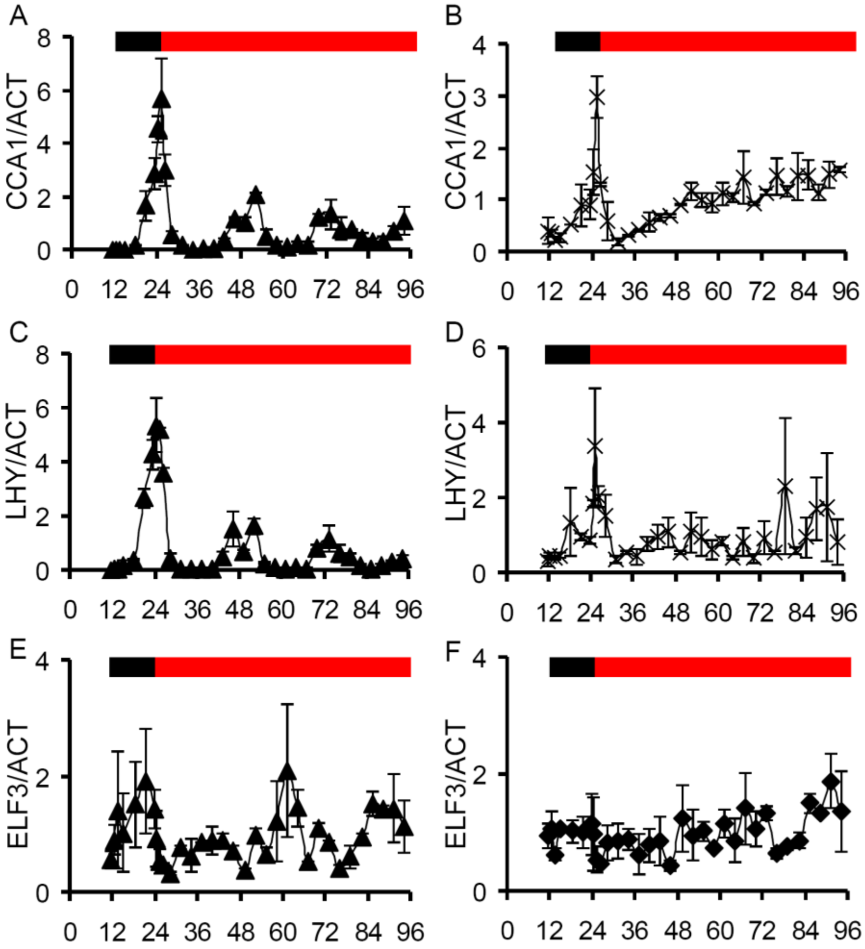

Figure S2 (related to Figure 1). Arrhythmic Expression of *CCA1*, *LHY* and *ELF3* in Clock Mutants

QPCR measurements of RNA levels from 12:12 red light:dark entrained 6 day old seedlings released into constant red light (13-20 µmol m^{-2} s^{-1}) for *CCA1* (A, B) , *LHY* (C,D) and *ELF3* (E,F), in (A, C, E,) Ws (filled triangles), (B,D) *elf3-4* (crosses) and (F) *cca1-11 lhy-21* (filled diamonds). Data are means and range of biological duplicates, with QPCR assays in technical triplicate for each sample. Expression levels are normalised to *ACTIN2*. In order to compare the expression waveforms, data have been normalised to the mean for each timeseries: note that therefore expression levels cannot be compared across panels. Relative levels in Ws and mutants are given in the main text.

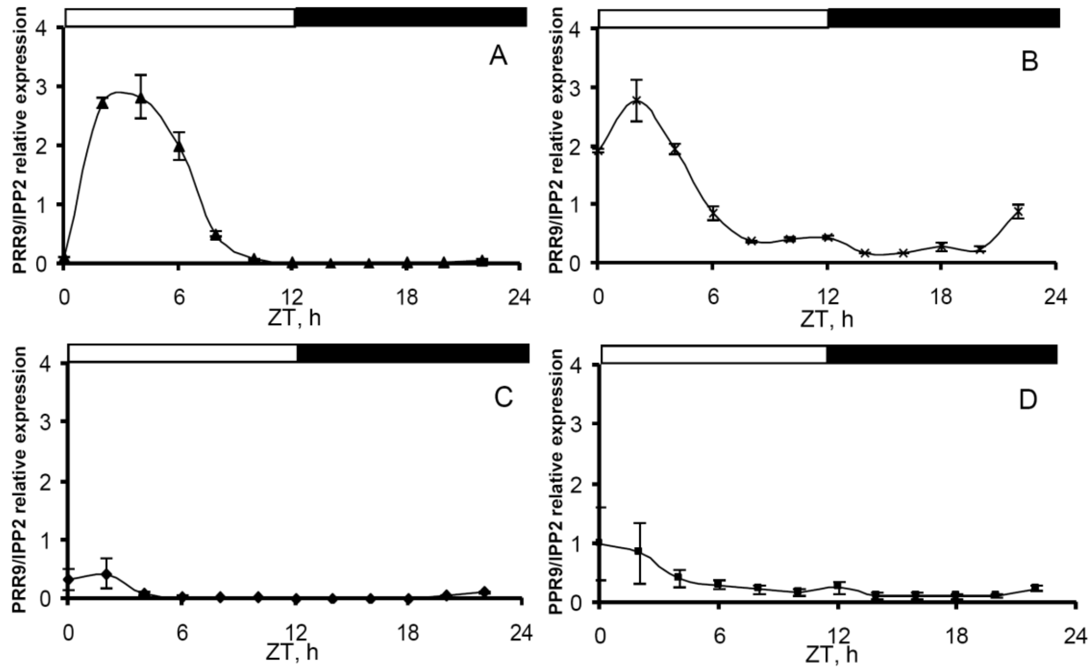
A
B
C
D
PRR9/IPP2 relative expression
PPR9/IPP2 relative expression
ZT, h
0
6
12
18
24
0
1
2
3
4

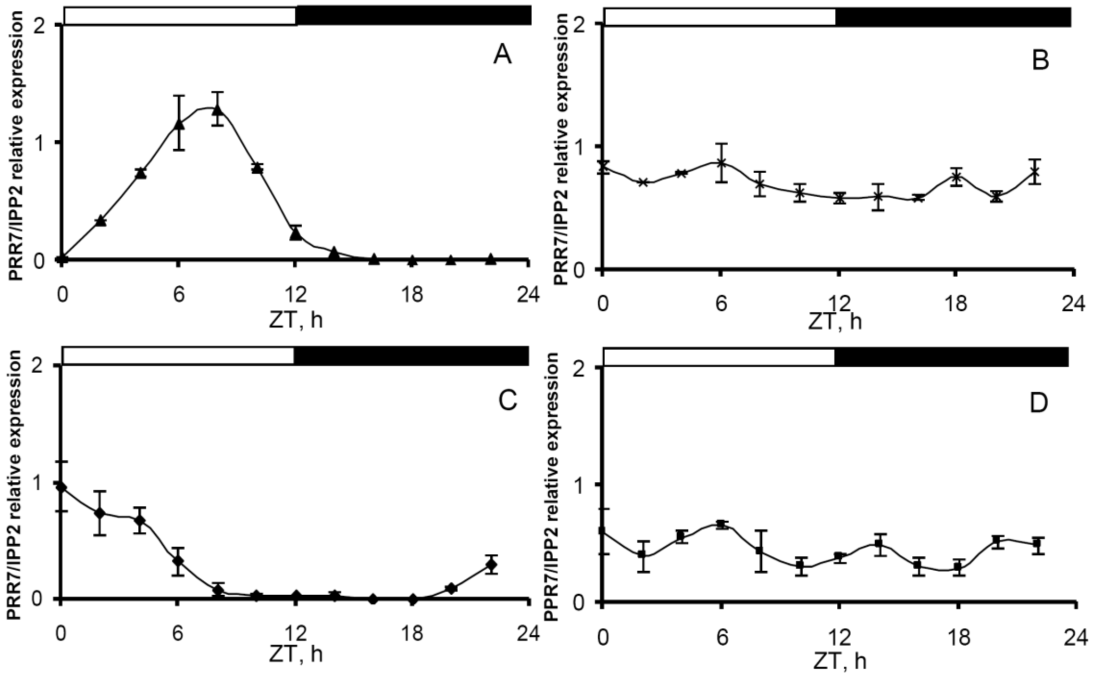
A
B
C
D
PRR7/IPP2 relative expression
PPR7/IPP2 relative expression
ZT, h
0
6
12
18
24
0
1
2

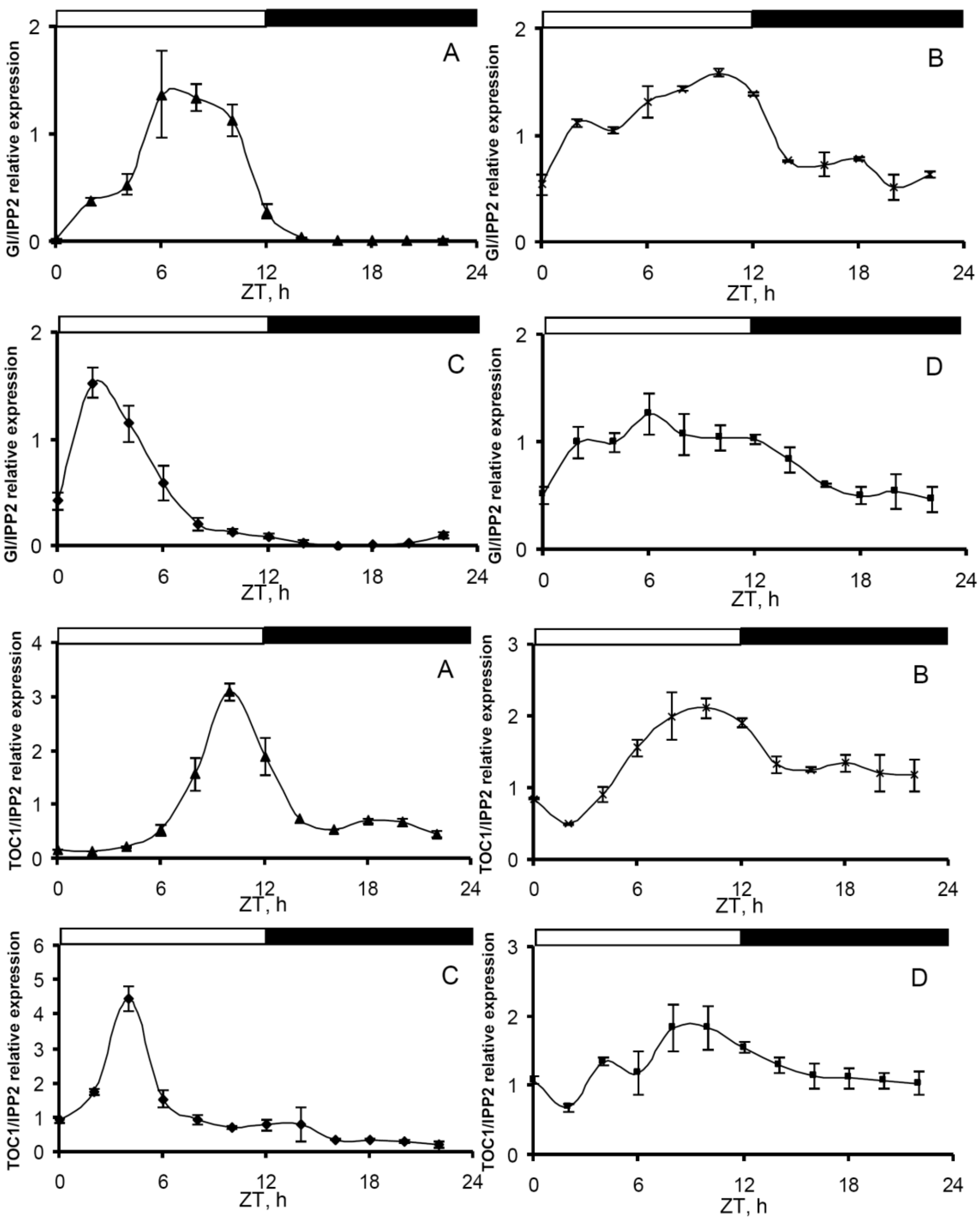

Figure S3 (Related to Figure 2). ELF3 Regulates the Expression of Core Circadian Genes
For clarity the results of Figure 2 are separated into individual plots for each gene (labelled on Y axis) and genotype (A- Ws; B – *elf3-4*; C - *cca1-11 lhy-21*; D - *cca1-11 lhy-21 elf3-4*). For further details, please see Figure 2 legend.

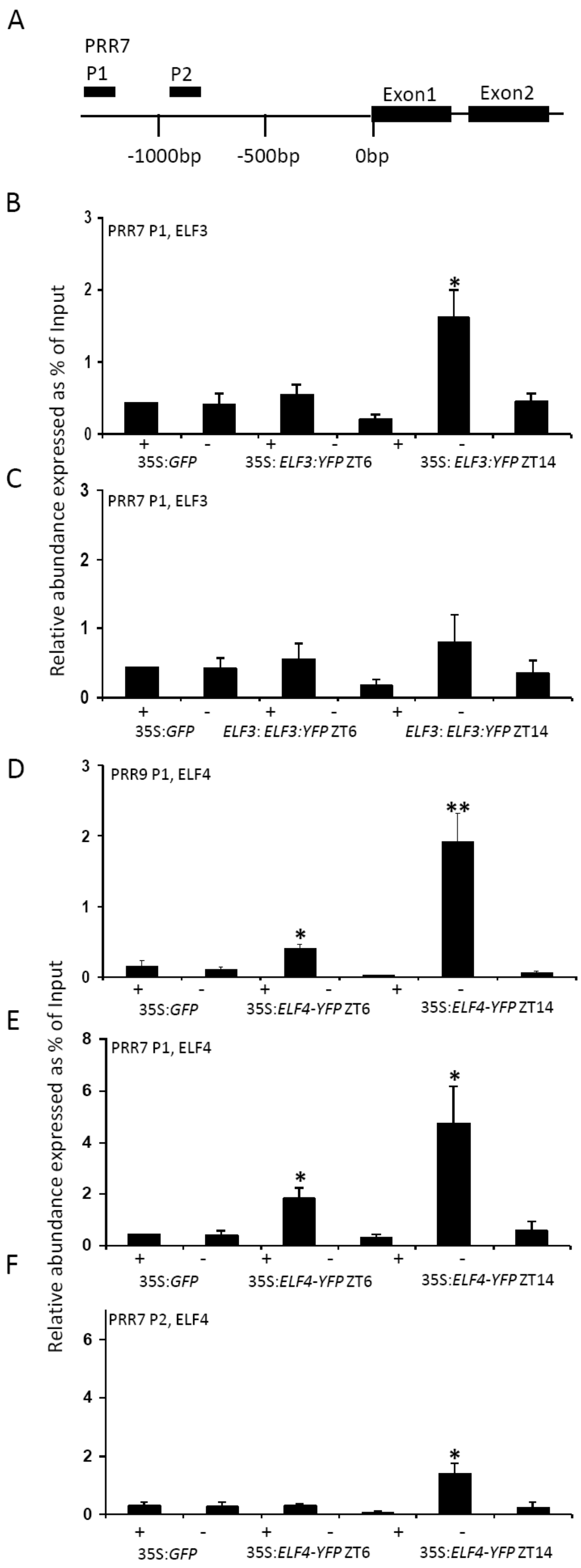
A
PRR7
P1
P2
Exon1
Exon2
-1000bp
-500bp
0bp
B
PRR7 P1, ELF3
35S:GFP
35S: ELF3:YFP ZT6
35S: ELF3:YFP ZT14
C
PRR7 P1, ELF3
35S:GFP
ELF3: ELF3:YFP ZT6
ELF3: ELF3:YFP ZT14
Relative abundance expressed as % of Input
D
PRR9 P1, ELF4
35S:GFP
35S:ELF4-YFP ZT6
35S:ELF4-YFP ZT14
E
PRR7 P1, ELF4
35S:GFP
35S:ELF4-YFP ZT6
35S:ELF4-YFP ZT14
F
PRR7 P2, ELF4
35S:GFP
35S:ELF4-YFP ZT6
35S:ELF4-YFP ZT14
Relative abundance expressed as % of Input

Figure S4 (Related to Figure 3). ELF3 Associates Only Weakly with the *PRR7* Promoter and ELF4 Can Also Bind In Vivo to *PRR7* and *PRR9* Promoter Regions

A) Schematic of the *PRR7* genomic region tested. The black bars indicate the location of the specific region amplified from ChIP DNA by primer sets PRR7 P1 and P2.

B-F) Chromatin of 3 week old seedlings was immunoprecipitated using either no antibody (-) or anti-GFP antibody(+). Resultant DNA from 35S:*GFP* (B-F), 35S:*ELF3:YFP* (B), *ELF3:ELF3:YFP* (C) and 35S:*ELF4:YFP* (D-F), was analysed by QPCR using PRR7 P1 (B, C & E), PRR9 P1 (D) and PRR7 P2 (F). Each signal is expressed as a percentage of the non-immunoprecipitated DNA (Input) extracted from the same tissue sample. Data represent the mean of at least 6 samples taken from 3 independent ChIP experiments. Error bars represent the SEM. Student's t-test was used to determine which samples had significantly different chromatin association from their no antibody control. Only samples showing a significant difference are marked *, $p<0.05$ or **, $p<0.005$.

Table S1. Molecular Markers Used to Detect *elf3-4, cca1-11* and *lhy-21* Mutations

Genotypes	Fragment Lengths (bp)		PCR Primers	
	Wild-Type Allele	Mutant Allele	Forward	Reverse
ELF3/*elf3-4*	11, 35, 83	11, 107	*elf3-4* dCAPS3F	*elf3-4* dCAPS3R
CCA1/*cca1-11*	1710	no product	CCA1F	CCA1R
	no product	~1300	CCA1F	JL270 LB primer
LHY/*lhy-21*	1671	no product	LHYF1	LHYR1
	no product	~1600	LHYR	JL270 LB primer

All markers are PCR-based and amplified fragments were resolved by agarose gel-electrophoresis.

Table S2. Primer Sequences Used in This Study

Sequence of primers used for genotyping *cca1-11*, *lhy-21* and *elf3-4* mutants:

***elf3-4* dCAPS3F:** 5'-AGGGCCTAGAGCTCCTCCTA-3'
***elf3-4* dCAPS3R:** 5'-CCAGGATGAACCAAAGTGCT-3'
CCA1F: 5'- AAAGCTGAATCATCTCTTCAGCCACTAGT-3'
CCA1R: 5'- GCTTGCGTTTGATGTCTCT-3'
JL270 LB primer: 5'-TTTCTCCATATTGACCATCATACTCATTG-3'
LHYF1: 5'-CTTACCAACGAAAGTAAGTCTAAGAAAGC-3'
LHYR1: 5'-AAAGATTGGAGAAGCAAACTACTAACACT-3'
LHYR: 5'- AACCTGACATGACCAAAGAAATGTTCGGA-3'

Sequence of primers used to amplify components of *ELF3:ELF3-YFP*, *35S:ELF3-YFP* and *35S:ELF4-YFP* constructs:

ELF3 promoter F (EcoRI): 5'-ATAGAATTCTGAATAACGCCAAACTTTTAGTGA-3'
ELF3 promoter R (SmaI): 5'-ATACCCGGGCACTCACAATTCACAACCTTTTTC-3'
ELF3 CDS F (EcoRV): 5'-CGGGATATCATGAAGAGAGGGAAAGATGAGG-3'
ELF3 CDS R (SmaI): 5'-ATTACCCGGGAGGCTTAGAGGAGTCATAGCG-3'
ELF4 CDS F (XbaI): 5'-CTTTCTAGAATGAAGAGGAACGGCGAGA-3'
ELF4 CDS R (SmaI): 5'-AAACCCGGGAGCTCTAGTTCCGGCAGCA-3'

Gene specific primer pairs used in QPCR

IPP2 F: 5'- GTATGAGTTGCTTCTCCAGCAAAG-3'
IPP2 R: 5'-GAGGATGGCTGCAACAAGTGT-3'
GI F: 5'-TATTGAAGTGTCGTCTACCAG-3'
GI R: 5'-GAGCTTTGGTTCATGATATCAC-3'
PRR9 F: 5'-GATTGGTGGAATTGACAAGC-3'
PRR9 R: 5'-TCCTCAAATCTTGAGAAGGC-3'
PRR7 F: 5'-CTTTCTCAAGGTATAATCCAGCC-3'
PRR7 R: 5'-ACAATCATATGCTGCTTCAGTC-3'
TOC1 F: 5'-ATCTTCGCAGAGTCCCTGTGATA-3'
TOC1 R: 5'-GCACCTAGCTTCAAGCACTTTACA-3'
ELF3 F: 5'-GGAAAGCCATTGCCAATCAA-3'
ELF3 R: 5'-ATCCGGTGATGCAGCAATAAGT-3'
CCA1 F: 5'-CTGTGTCTGACGAGGGTCGAA-3'
CCA1 R: 5'-ATATGTAAAACTTTGCGGCAATACCT-3'
LHY F: 5'-CAACAGCAACAACAATGCAACTAC-3'
LHY R: 5'-AGAGAGCCTGAAACGCTATACGA-3'

Primer pairs used in ChIP

PRR9 1F: 5'-GCCGCGATACAGAGAAAATC-3'
PRR91R: 5'-TTCGATCACAACCACGAAA-3'

PRR9 2F: 5’-CGCCCACTAACGAAATTTGA-3’
PRR9 2R: 5’-TTCAAATTGGATGGCTTTTT-3’
PRR9 3F: 5’-GGATCTTTTTCTTCGTCAATGG-3’
PRR9 3R: 5’-TGATGTGGACAGTGCGTAAT-3’
TOC1 1F: 5’-TCTCCGGTGACTTTTGTTGA-3’
TOC1 1R: 5’-TGGCCAAATCAGAACTAGGG-3’
TOC1 2F: 5’-CGTCATCTCCTTGGCCTAAA-3’
TOC1 2R: 5’-CGGTGAGATGAGGAGGAGAG-3’
TOC1 3F: 5’-GGAGTCTTTTGTGATGAGAAAATTG-3’
TOC1 3R: 5’-GACCAAACCATCAGAAACCAA-3’
TOC1 4F: 5’-GGTCGATGGCAAAACGTAAT-3’
TOC1 4R: 5’-TCATCAGTGGTTGGGAAACA-3’
TOC1 5F: 5’-AAGCCGCCAAATATAAACCA-3’
TOC1 5R: 5’-AAATGCCCTTTTCAGACACG-3’
GI 1F: 5’-CGGATGAAAACTAAACCAGCA-3’
GI 1R: 5’-CAGAAGGACCGTGACATCAA-3’
GI 2F: 5’-TCTCGTTTGCTAAACCACAAAA-3’
GI 2R: 5’-AAAAGCTACTTTGCCTACCTCTTT-3’
GI 3F: 5’-ATCTTATTGCGCCCACGTCTC-3’
GI 4F: 5’-ACCACCAAACCTTGAAATAAAA-3’
GI 4R: 5’-CCAAGAAAAATGTTTGCCAAT-3’
GI 5F: 5’-TCTCTCTCCTAAGGCCACCA-3’
GI 5R: 5’-CAATCAACCAAAAACCACGA-3’
GI 6F: 5’-TTTCGTGGTTTTTGGTTGA-3’
GI 6R: 5’-CGAAGCTGAATCAAACAGCTAA-3’
PRR5 1F: 5’-GCCGGCCTAAACCTATTTCT-3’
PRR5 1R: 5’-TGACTGGACCCTATGGTTTTC-3’
PRR5 2F: 5’-ATCCACCAAAGAACCACGAG-3’
PRR5 2R: 5’-ATCAAATGGAACATGCACGA-3’
PRR5 3F: 5’-CCATGTGTCCTTGATTTTCTTATG-3’
PRR5 3R: 5’-ATCCCACTCGTGACTTTTGG-3’
CCA1 1F: 5’-ACCCTTCATGCATGGTTAGC-3’
CCA1 1R: 5’-CATTCTCGTGCGGTTCACTA-3’
CCA1 2F: 5’-GTCGACAAACTGGTGGGAGA-3’
CCA1 2R: 5’-TCCGGGACTACCTGAAAGG-3’
CCA1 3F: 5’-TTCGTCTGGAGAAGATCTGG-3’
CCA1 3R: 5’-GTCCACCTTTCACGTTGCTT-3’
PRR7 1F: 5’-GCGTGAAGGAACACTGAAGG-3’
PRR7 1R: 5’-ACGACGTTATCACGGAGCTT-3’
PRR7 2F: 5’-TGTCGATATGTCCGAGTGGT-3’
PRR7 2R: 5’-GGTGGGTAAGGAAAACGTCA-3’
PRR7 3F: 5’-GGGTTTATGGCTGTGTTTTGA-3’
PRR7 3R: 5’-CTCCGGTCTTTCGATCAGTG-3’

Supplemental References

S1**.** Gould, P. D., Diaz, P., Hogben, C., Kusakina, J., Salem, R. Hartwell, J. and Hall, A. (2009). Delayed fluorescence as a universal tool for the measurement of circadian rhythms in higher plants. Plant J., 58, 893-901.

S2. Plautz, J. D., Straume, M., Stanewsky, R., Jamison, C. F., Brandes, C., Dowse, H. B., Hall, J. C. and Kay, S. A. (1997). Quantitative analysis of Drosophila period gene transcription in living animals. J. Biol. Rhythms, 12, 204-217.

S3. Pokhilko, A., Hodge, S.K., Stratford, K., Knox, K., Edwards, K.D., Thomson, A.W., Mizuno, T., Millar, A.J. (2010). Data assimilation constrains new connections and components in a complex, eukaryotic circadian clock model. Mol. Syst. Biol., 6: 416.
